# The Very Low-Dose Dexamethasone Suppression Test in the General Population: A Cross-Sectional Study

**DOI:** 10.1371/journal.pone.0164348

**Published:** 2016-10-13

**Authors:** Nese Direk, Marieke J. H. J. Dekker, Annemarie I. Luik, Clemens Kirschbaum, Yolanda B. de Rijke, Albert Hofman, Witte J. G. Hoogendijk, Henning Tiemeier

**Affiliations:** 1 Department of Epidemiology, Erasmus Medical Centre, Rotterdam, the Netherlands; 2 Department of Psychiatry, Dokuz Eylul University, Izmir, Turkey; 3 Department of Internal Medicine, VU Medical Centre, Amsterdam, the Netherlands; 4 Sleep and Circadian Neuroscience Institute, Nuffield Department of Clinical Neurosciences, University of Oxford, Oxford, United Kingdom; 5 Department of Biological Psychology, Technical University of Dresden, Dresden, Germany; 6 Department of Clinical Chemistry, Erasmus Medical Centre, Rotterdam, the Netherlands; 7 Department of Psychiatry, Erasmus Medical Centre, Rotterdam, the Netherlands; 8 Department of Child and Adolescent Psychiatry, Erasmus Medical Centre, Rotterdam, the Netherlands; Tel Aviv Sourasky Medical Center, ISRAEL

## Abstract

Determinants of the hypothalamic-pituitary-adrenal (HPA) axis functioning are increasingly explored in population-based studies. However, functional tests measuring the negative feedback of the HPA axis cannot easily be implemented into large observational studies. Furthermore, high doses of dexamethasone often completely suppress the HPA axis in healthy persons. This study aimed to detect the effects of the health, lifestyle and sociodemographic factors, psychiatric problems and cognitive functions on the negative feedback of the HPA axis using a very low-dose (0.25 mg) dexamethasone suppression test (DST).We evaluated the associations of several determinants with the saliva cortisol concentrations after dexamethasone intake in a confounder-adjusted model also corrected for baseline saliva cortisol concentrations in the Rotterdam Study, a large population-based study (N = 1822). We found that female sex, low income, lack of exercise, instrumental disability and smoking were all independently associated with stronger suppression of the HPA axis. Even though there were no linear associations between psychiatric measures and cortisol suppression, we found that depressive symptoms and anxiety disorders were more common in persons with non-suppression of cortisol. Conversely, psychotropic medication use was related to enhanced suppression of cortisol after DST. In this large study, we found that female gender, low socioeconomic status and poor health were all related to suppression of the HPA axis. Non-linear associations were detected between the suppression of the HPA axis and common psychiatric disorders in community-dwelling persons.

## Introduction

The hypothalamic-pituitary-adrenal (HPA) axis controls the stress response in the body. To explain the relation between diurnal HPA axis functioning and metabolic or stress-related disorders in large cohorts of healthy persons, researchers relied on single morning blood measures or used repeated saliva sampling. Different measures of the HPA axis such as cortisol awakening response, diurnal decline and total cortisol exposure over the day have been assessed using mostly saliva [1, 2]. More recently, cortisol assessment in hair became possible [3]. In contrast, negative feedback of the HPA axis is rarely tested in non-clinical studies [4, 5].

In clinical populations, the negative feedback of the HPA axis is assessed by a dexamethasone suppression test (DST) requiring oral administration of a dose of dexamethasone. Cortisol secretion after dexamethasone intake is typically suppressed. A non-suppression indicates hypercortisolemia [6] but DST proved very difficult to implement in population-based studies.

The DST was originally designed to diagnose patients with Cushing’s Syndrome using 1 mg or higher doses of dexamethasone with a cut-off value for the plasma cortisol to distinguish between non-suppressors and suppressors. A dose of 1 mg in the conventional DST fully suppresses the HPA axis in most persons from a non-clinical population [7]. Furthermore, the traditional “black and white” outcome definition of the negative feedback of the HPA axis masks any variation across the continuum of HPA axis reactivity. To this aim, cut-points were defined for the conventional DST, which relies on plasma cortisol to define suppression. In this population-based study, we tested the determinants of the negative feedback of the HPA axis with a very low-dose DST (0.25 mg) and assessed the outcome (suppression) continuously.

Different determinants of the suppression or non-suppression after DST have been explored in the last decades. Over the years, the DST became perhaps the most common function test in clinically depressed persons [2, 7–10]. However, like in other psychiatric disorders including post-traumatic stress disorders, anxiety disorders, eating disorders, psychosis, personality traits, results of this test are inconsistent [11–19].

The majority of research has been performed in clinical samples with small sample sizes. Clinical studies are generally more efficient to test associations of an exposure and disease than population-based cohort studies. However, evaluating background variables is generally not possible in clinical studies. Often, only few background variables are assessed and background variables are related to disease (case) status, which gives rise to selection bias. Sometimes if matching is performed in clinical studies to control for the effect of a potential confounder. It is not possible to explore the importance of this matching variable for cortisol suppression anymore. Yet, evaluating determinants and potential confounders of the negative feedback of the HPA axis is important as numerous psychological and physiological stressors can potentially affect this system. Also, knowledge about the possible confounders of the cortisol suppression is mostly derived from the measurement of single cortisol levels or diurnal cortisol secretion. Thus, large population-based studies are needed to explore the determinants of the reaction to the DST. Until now, only one cohort explored the determinants (non-psychiatric) of negative feedback of the HPA axis using the low-dose DST (0.5 mg dexamethasone) [5]. In this study (n = 455), a non-suppression of cortisol in the low-dose DST was observed in smokers, less active persons and sampling on a weekday. However, 86.4% of the study population was below the study-specific cut-off for cortisol suppression suggesting that 0.5 mg might be a too high dose to evaluate functioning of the HPA axis in the general population.

Here, we aimed to explore the most important determinants of the negative feedback sensitivity of the HPA axis using a very low-dose (0.25 mg dexamethasone) DST in a large population-based study evaluating several determinants of health, lifestyle and sociodemographic factors, psychiatric problems and cognitive functions.

## Materials and Methods

The Rotterdam Study is a prospective population-based cohort investigating occurrence and determinants of chronic diseases. It has been approved by the institutional review board of the Erasmus University Medical Center and by the review board of The Netherlands Ministry of Health, Welfare and Sports. All participants provided written informed consent after complete description of the Rotterdam Study.

The current study was embedded in the third cohort of the Rotterdam Study (RS-III), which was initiated in 2006 and ended in 2008. Details regarding the study design of the RS-III are provided elsewhere [20]. This cohort included 3,932 subjects aged 45 to 54 years and those who had moved to the study district regardless of age. Among those, 3,247 (82.6%) participated in the day-curve saliva cortisol collection. We invited these participants for the DST and 2076 (63.9%) agreed to also take a dexamethasone tablet and to collect two additional saliva samples. Fifty-eight participants did not report time of sampling of a saliva sample, and 130 did not produce enough saliva to analyze cortisol. Further, we excluded participants from all analyses if the time difference between the two samples deviated more than 3 h from the specified 24h (n = 59) (see sensitivity analysis for more stringent exclusion criteria based on sampling interval). Finally, we excluded participants, who reported the use of systemic corticosteroids (n = 7) leaving 1822 participants for the current study. When compared to the participants (n = 1822), non-participants who did not participated in the DST or were excluded from the analyses (n = 1425) were more likely to be male (p = .007) and younger (p < .001).

### Assessment of the Dexamethasone Suppression Test

Participants were asked to collect saliva samples at home using the Salivette sampling devices (Sarstedt, Nümbrecht, Germany). They received detailed oral and written instructions with particular emphasis on the importance of compliance to sampling time. Participants were instructed to collect the first saliva sample at 8 am, to take the dexamethasone pill at 11 pm at the same day, and to collect the second saliva sample the next morning at 8 am. This approach was chosen as similar sampling times have commonly been used in clinical and population-based studies. High early morning levels of cortisol maximize the power to detect differences [2]. Additionally participants were instructed not to eat and not to brush their teeth 15 minutes before collecting the samples and to report the exact time and date of the sampling and dexamethasone intake on a form provided by the researchers. More detail about the instructions given to the participants are provided in the supplement.

We kept the Salivettes at -80°C until they were sent to the Laboratory of Biopsychology, Technical University of Dresden, Germany. Salivary cortisol concentrations were measured using a commercial immunoassay with chemiluminescence detection (CLIA; IBL Hamburg, Hamburg, Germany). Intra-assay and interassay coefficients of variation were less than 6% and 9%, respectively (obtained for control samples with average cortisol levels 4.1 nmol/l and 29.5 nmol/l). The lower limit of detection was 0.4 nmol/liter. Saliva cortisol levels were below the detection limit in one person for the second saliva sample in our sample. A level of zero nmol/l was used for this participant. Before dexamethasone intake, none of the participants had a cortisol level below the detection limits in the current study sample.

### Assessment of Determinants

We selected possible determinants of cortisol suppression after DST on the basis of previous literature and grouped them into four categories similar to the previous epidemiological studies: Sociodemographic indicators, health and lifestyle variables, psychiatric problems and cognitive functions and sampling variables [1, 5].

Sociodemographic indicators were age, gender, education and monthly household income. Educational attainment was assessed in seven categories from primary education to university level on the basis of the Standard Classification of Education. In this study, we grouped education into three categories including low, intermediate and high. Monthly net household income was reported in 13 ordinal categories (from < 500 € to ≥ 2900 €) and analyzed continuously.

We studied body mass index, smoking status, disability, exercise, diabetes mellitus and hypertension as indicators of health and lifestyle. Height (meters) and weight (kilograms) were measured and body mass index was calculated as kg/m^2^. Smoking status was coded in three categories as never, former, and current smoker. Different dimensions of disability were evaluated using Activities of Daily Living (ADL) from the Stanford Health Assessment Questionnaire [21] and Instrumental Activities of Daily Living (iADL) [22] scales. We used a predefined cut-off ADL score of 0.5 to define a mild or severe disability [23]. A cut-off score of 0.23, which is one standard deviation of the mean iADL score was used to define persons with instrumental disability. Exercise was evaluated using a single item from a questionnaire that was prepared by researchers evaluating sport habits on a regular basis. Diabetes mellitus was diagnosed if participants were on antidiabetic medication or if their fasting blood glucose concentrations were 200 mg/dL or higher. Systolic and diastolic blood pressures were measured twice in the resting state with a random-zero sphygmomanometer and the mean of two consecutive measurements was calculated. Hypertension was defined as a systolic blood pressure ≥140 mm Hg or a diastolic blood pressure ≥90 mm Hg or the use of antihypertensive medication.

We examined general cognitive functions, clinically relevant depressive symptoms, depressive disorders, anxiety disorders, psychotic experiences, and psychotropic medication as psychiatric variables. General cognitive functions were measured with the Mini–Mental State Examination (MMSE) [24] and a g-factor. This g-factor was derived from principal component analysis of processing speed, executive function, verbal fluency, verbal recall and recognition, visuospatial ability and fine motor skills [25]. Persons with an MMSE score of less than 24 were categorized as cognitively impaired. Depressive symptoms were evaluated with the Center for Epidemiological Studies-Depression (CES-D) scale and a cut-off of 16 was used to detect participants with clinically relevant depressive symptoms [26, 27]. All participants with clinically relevant depressive symptoms were invited for a semi-structured clinical interview, the Schedules for Clinical Assessment in Neuropsychiatry (SCAN) [28] to detect DSM-IV-TR diagnosis of depressive disorders. These interviews were performed by trained clinicians and psychologists in close proximity in time to screening (on average 2 weeks). Anxiety disorders were diagnosed with an adapted version of the Munich version of the Composite International Diagnostic Interview (M-CIDI) [29, 30]. All DSM-IV based anxiety disorders (except obsessive-compulsive disorder and post-traumatic stress disorder) were assessed and studied as a categorical variable. Psychotic experiences were evaluated with the Community Assessment of Psychic Experiences (CAPE)-positive experiences scale (20 self-reported items) [31, 32]. Each item assesses frequency and distress related to the psychotic experience. Data on psychotropic medication use was obtained from pharmacy records.

As sampling variables, we considered cortisol concentrations before dexamethasone intake and the day of the sampling. The day of the sampling was categorized as weekday or weekend. In addition, all analyses were adjusted for the times of saliva sampling before and after dexamethasone intake.

### Statistical Analyses

We used the natural-log function to transform the cortisol concentrations after dexamethasone intake because of the skewed distribution (Figures A and B in S1 File). We present effect estimates only for the transformed values. First, we examined the relation of the individual determinants with cortisol concentrations after dexamethasone intake using linear regression analysis. The basic model was adjusted for age, sex and cortisol concentrations assessed the previous day. This adjustment is considered a more reliable method than change score (cortisol after- cortisol before) and percentage change ([cortisol after- cortisol before / cortisol before]*100) methods. Moreover, a simulation study showed that analysis using percentage change has very low statistical power when compared to the model in which a post-test value is used as an outcome and a pre-test value is used as a confounder. [33, 34]. Second, we fitted a multivariable model in which all determinants used in the current study were included in the same model. Because of the well-known effect of body weight on the associations of gender and smoking status with cortisol, these analyses (both basic and multivariable models) were additionally adjusted for body weight.

In addition, non-linear associations of psychiatric traits were explored contrasting the highest and lowest tenth percentiles versus the middle group. The highest tenth percentile was used to define participants with non-suppression and the lowest tenth percentile was used to define participants with enhanced suppression.

All analyses were further adjusted for time of the first and second cortisol samplings.

In addition, we performed sensitivity analyses to further test the possible effect of the time interval between the first and second cortisol sampling. We reran the multivariable model including only participants who complied fully with a time interval of 24 ± 1 hours (n = 1632) between the moments of saliva sampling and also including only those with a time interval of 24 ± 2 hours (n = 1766).

In a supplementary analysis, we tested the associations of the selected variables with the baseline cortisol concentrations (i.e., the concentrations before dexamethasone intake) to explore the associations with the non-suppressed basal cortisol secretion. These contrasting analyses help to evaluate which findings of the very-low dose dexamethasone test are specific for cortisol suppression.

To facilitate the comparison of the effect estimates of the various variables independent of the units of variables, we reported the standardized regression coefficients. All determinants had less than 10% of missing values. To enhance the comparability across the results, we imputed missing values of the determinants using the Expectation-Maximization Algorithm.

SPSS version 20 (SPSS, Inc., Chicago, IL) was used for all analyses.

## Results

**Fig 1:**
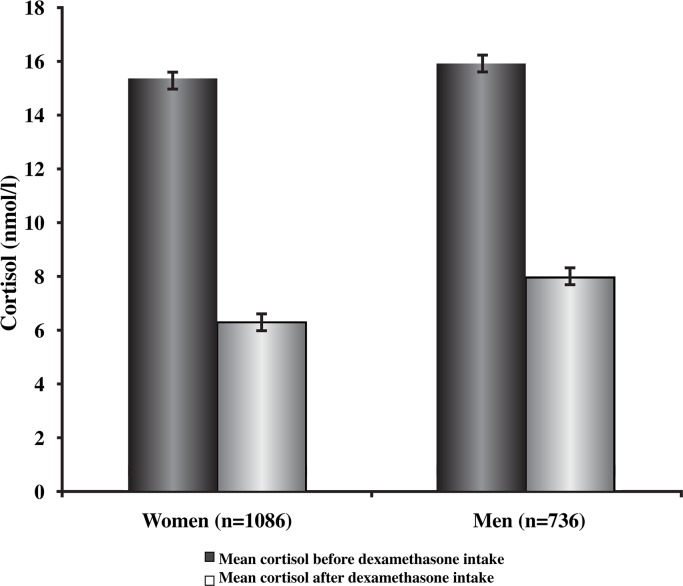
Cortisol concentrations before and after dexamethasone intake. Cortisol concentrations were significantly lower after dexamethasone intake in both men (*p <* .001) and in women (*p <* .001). The difference in cortisol concentrations before dexamethasone intake between women and men were not significantly different (*p* = .23). In contrast, there was a significant difference in cortisol concentrations after dexamethasone intake between women and men (*p <* .001).

The participant characteristics are presented in Table 1. The mean age was 57.8 years (standard deviation [SD] = 6.8 years) and 59.6% (n = 1086) of the study population was female. The mean cortisol concentration before dexamethasone intake was 15.6 nmol/L (SD = 10.9) and the mean concentration after dexamethasone intake was 7 nmol/L (SD = 8.5). Gender specific mean cortisol concentrations before and after dexamethasone intake are shown in Fig 1.

**Table 1:** Characteristics of the study population.

Characteristics	Study sample N = 1822
**Sociodemographic indicators**	
Age, years, mean (SD)	57.8 (6.8)
Women, n (%)	1086 (59.6)
Low education, n (%)	183 (10)
Paid job, n (%)	975 (53.5)
Net income, median (range)	11^a^ (1–13)
Married, n (%)	1433 (78.6)
**Health & lifestyle variables**	
Mild/severe disability, n (%)	313 (17.2)
Instrumental disability, n (%)	215 (11.8)
Regular exercise, n (%)	1104 (60.6)
Smoking status, n (%)	
Current smoker	377 (20.7)
Former smoker	847 (46.5)
Never smoker	598 (32.8)
Body mass index, kg/m^2^, mean (SD)	27.7 (4.4)
Diabetes mellitus, n (%)	158 (8.7)
Hypertension, n (%)	901 (49.5)
**Psychiatric problems & cognitive functions**	
Depressive symptom score, mean (SD)	5.7 (7.3)
Clinically relevant depressive symptoms, n (%)	180 (9.9)
Major depressive disorder, yes, n (%)	31 (1.8)
Anxiety disorders, n (%)	148 (8.1)
MMSE score, mean (SD)	28.1 (1.9)
Cognitive impairment, yes, n (%)	37 (2)
The *g*-factor of the cognitive tests, mean (SD)	0.02 (0.99)
Psychotic experiences (CAPE-positive items) frequency score, mean (SD)	1.1 (0.12)
Psychotropic medication, yes, n (%)	270 (14.9)
**Sampling variables**	
Day of the sampling, weekday, n (%)	1784 (97.9)
Cortisol before dexamethasone intake, nmol/L, mean (SD)	15.6 (10.9)
Time of the first sampling, hh:mm, mean (SD)	07:58 (00:44)
Cortisol after dexamethasone intake, nmol/L, mean (SD)	7.0 (8.5)
Time of the second sampling, hh:mm, mean (SD)	07:53 (00:37)

*Notes*: Imputed values are presented. *Abbreviations*: MMSE, Mini-Mental State Examination; CAPE, The Community Assessment of Psychic Experience

^a^ 11 represents monthly household income between 2100–2500 Euro.

Table 2 shows the results of the associations between determinants and the negative feedback control of cortisol as indicated by cortisol concentrations after the very low-dose DST adjusted for baseline cortisol concentration. The baseline cortisol concentrations before dexamethasone intake were substantially and positively associated with cortisol concentrations after dexamethasone intake (*β =* 0.41, p < .001); i.e., high cortisol concentrations before dexamethasone intake were related to high cortisol concentrations after dexamethasone intake.

**Table 2:** Relations of determinants with cortisol concentrations after dexamethasone intake.

	Cortisol concentration after dexamethasone intake							
	Basic model^a^					Mutually adjusted model^b^			
Sociodemographic indicators	*β*	B	95% CI	*p*	*β*	B	95% CI	*p*
Age (years)	-0.05	-0.01	-0.01; -0.001	.017	-0.02	-0.002	-0.01; 0.004	.43
Sex (0 = male, 1 = female)	-0.16	-0.28	-0.35; -0.21	< .001	-0.16	-0.27	-0.35; -0.20	< .001
Education								
Low	-0.01	-0.02	-0.15; 0.11	.71	0.02	0.07	-0.07; 0.20	.32
Intermediate	-0.05	-0.08	-0.16; 0.001	.052	-0.01	-0.02	-0.11; 0.06	.58
High		(reference)				(reference)		
Net income (ranked 1 to 13)	0.08	0.02	0.01; 0.04	.001	0.06	0.02	0.003; 0.03	.020
**Health & lifestyle variables**								
Mild/severe disability (0 = no, 1 = yes)	-0.03	-0.06	-0.15; 0.04	.24	-0.02	-0.04	-0.14; 0.05	.36
Instrumental disability (0 = no, 1 = yes)	-0.07	-0.18	-0.29; -0.07	.001	-0.07	-0.17	-0.28; -0.06	.002
Regular exercise (0 = yes, 1 = no)	-0.08	-0.13	-0.20; -0.06	< .001	-0.05	-0.09	-0.16; -0.01	.019
Smoking								
Never smoker		(reference)				(reference)		
Former smoker	0.02	0.03	-0.05; 0.11	.43	0.02	0.03	-0.05; 0.11	.48
Current smoker	-0.09	-0.19	-0.28;-0.09	< .001	-0.07	-0.15	-0.25; -0.05	.002
Body mass index (kg /m^2^)	0.01	0.002	-0.01; 0.01	.70	0.01	0.002	-0.01; 0.01	.60
Diabetes mellitus (0 = no, 1 = yes)	-0.04	-0.11	-0.23; 0.02	.09	-0.03	-0.08	-0.21; 0.04	.20
Hypertension (0 = no, 1 = yes)	0.02	0.03	-0.04; 0.10	.37	0.02	0.03	-0.04; 0.10	.39
**Psychiatric problems & cognitive functions**								
Depressive symptom score	-0.10	-0.001	-0.01; 0.004	.63	0.02	0.003	-0.003; 0.01	.32
Clinically relevant depressive symptoms (0 = no, 1 = yes)	0.001	0.003	-0.11; 0.12	.96	0.03	0.08	-0.04; 0.20	.21
Major depressive disorder (0 = no, 1 = yes)	0.02	0.14	-0.12; 0.40	.29	0.04	0.24	-0.04; 0.51	.09
Anxiety disorders (0 = no, 1 = yes)	0.01	0.04	-0.08; 0.17	.49	0.02	0.06	-0.07; 0.19	.39
MMSE score	0.03	0.01	-0.003; 0.03	.11	0.01	0.01	-0.01; 0.02	.56
Cognitive impairment (0 = no, 1 = yes)	-0.01	-0.05	-0.29; 0.19	.68	0.01	0.04	-0.21; 0.28	.77
The *g*-factor of the cognitive tests	0.07	0.06	0.02; 0.10	.01	0.03	0.03	-0.02; 0.07	.23
Psychotic experiences (CAPE-positive items) frequency score	-0.04	-0.03	-0.63; 0.13	.19	-0.02	-0.13	-0.51; -0.26	.52
Psychotropic medications (0 = no, 1 = yes)	-0.04	-0.10	-0.19; 0.001	.05	-0.03	-0.06	-0.16; 0.04	.23
**Sampling variables**								
Day of the sampling (0 = weekend, 1 = weekday)	-0.004	-0.02	-0.26; 0.21	.85	-0.01	-0.04	-0.28; 0.19	.73
Cortisol before dexamethasone intake, nmol/L	0.40	0.03	0.03; 0.03	< .001	0.41	0.03	0.03; 0.03	< .001

^a^Adjusted for cortisol concentrations before dexamethasone intake, age and sex

^b^ Mutually adjusted model when appropriate.

*Abbreviations*: *β*, standardized beta; B, unstandardized beta; CI, Confidence Interval of the unstandardized beta; MMSE, Mini-Mental State Examination; CAPE, The Community Assessment of Psychic Experience

Cortisol suppression after dexamethasone intake was higher in women when compared to men (*β* = -0.16, p < .001). As women were lighter than men (74.38 kg, SD = 13.77 vs. 88.12 kg, SD = 13.4), we corrected the basic and mutually adjusted models for body weight. However, the gender difference in cortisol levels remained significant (results not shown). Older age was also associated with stronger cortisol suppression after dexamethasone intake (*β* = -0.05, p = .017), but this association was not significant in the multivariable model in which several health-related variables were included. Low income was related to stronger cortisol suppression after very low-dose DST in the multivariable model (*β* = 0.06, p = .020). Indicators of poor health such as instrumental disability (*β* = -0.07, p = .002), lack of exercise (*β* = -0.05, p = .019) and smoking (*β* = -0.07, p = .002) were all related to increased negative feedback control of cortisol in the multivariable models. To eliminate the possible effect of weight on the association of smoking status and cortisol suppression, we adjusted the basic and mutually adjusted models for weight instead of BMI; results remained significant (results not shown). Poor global cognition function (*β* = 0.07, p = .01) was related to stronger cortisol suppression after dexamethasone intake in the basic model. However, this association was not significant in the multivariable model. Use of psychotropic medications was related to enhanced cortisol suppression following dexamethasone intake in the age- and gender adjusted model (*β* = 0.04, p = .05). This association disappeared after adjustment in the multivariable model. Clinically relevant depressive symptoms, depressive disorders, anxiety disorders, psychotic experiences were not related to the negative feedback control of cortisol in any of the models. To test non-linear associations of psychiatric traits with cortisol suppression, we defined non-suppression and enhanced cortisol suppression patterns. Depressive symptom scores (OR = 1.02, *p* = .03), clinically relevant depressive symptoms (OR = 1.93, *p* = .01) and anxiety disorders (OR = 2.42, *p =* 0.001) were related to non-suppression. In contrast, psychotropic medication use was related to enhanced cortisol suppression (OR = 2.08, *p*< .001) (Table B in S1 File).

The multivariate model explained 22% of the variance (*F* (18, 1803) = 29.46, p < .001).

Adjustment for time of sampling did not change the results in any of the models (data not shown). Day of the sampling was not associated with cortisol suppression after dexamethasone intake.

To contrast the results with the determinants of basal cortisol secretion, we also examined the relation of these variables with cortisol concentration before dexamethasone intake. In this analysis, a different pattern of associations emerged: instrumental disability, BMI and cognition were all related to basal cortisol concentrations (Table A in S1 File). However, sex, smoking, low income and lack of exercise were not associated with basal cortisol levels. In summary, only instrumental disability determined both the degree of cortisol suppression and basal cortisol concentrations.

The sensitivity analyses excluding poor self-reported compliers suggests that excluding people with poor compliance to the DST protocol did not change the effect estimates meaningfully (Figure C in S1 File).

## Discussion

In this large population-based study, we found that female sex, low income, lack of exercise, instrumental disability and smoking were all associated with stronger suppression of the HPA axis as determined by lower cortisol concentrations after a very low-dose DST.

We observed that women had stronger suppression of the HPA axis than men. Our result is consistent with a previous report in which an increased negative feedback control of cortisol was found in women without psychopathology [5]. This gender difference might be related to circulating gonadal steroids in women. The mechanisms are not well understood; it is known that high concentrations of estradiol may alter the negative feedback control of cortisol leading to high cortisol levels [35]. In contrast, a decrease in estradiol levels during the menopause, as probably experienced by the women in our study (mean age of 57.8 years), may cause stronger suppression of the HPA axis [36, 37]. The gender difference might also results from weight differences as, on average, women are lighter than men. This difference could explain a greater effect of dexamethasone in lean people (i.e. women in our study) leading to more cortisol suppression. However, our results did not change when we adjusted for body weight. Low income, an indicator of low SES, was also associated with stronger suppression of the HPA axis. It is known that ongoing financial strain may give rise to chronic stress [38] which has repeatedly been linked to stronger suppression of the HPA axis. Low income has previously been associated with high [39–41] and low cortisol concentrations in studies with single time-point cortisol sampling [42]. However, our results cannot be compared straightforwardly with the findings of these studies, because income was not tested in relation to the negative feedback of the HPA axis in the general population.

Health and lifestyle variables including physical disability, lack of exercise, and smoking were associated with increased negative feedback sensitivity. Physical activity is considered as a direct stimulator of the HPA axis [43]. In a previous population-based study, however, physical activity as measured by metabolic equivalent (MET) of the number of calories expended per minute in an activity and cortisol level after dexamethasone intake were not related. In the same study, less MET was related with lower cortisol suppression ratio indicating a non-suppression [5]. In the current study, the questions on physical activity addressed whether a person performed at least one sport activity on a regular basis. Like in most measures, a positive answer indicates physical activity and fitness but may also reflect general health. Good health is a prerequisite for sports participation and this may contribute to the inconsistency between two studies.

Smoking has been associated with a hyperactive HPA axis in previous population-based studies [5, 44, 45], although habitual smokers have low basal cortisol concentrations. Habitual smokers develop desensitization to nicotine exposure at the acetylcholinergic receptor level. Unless the nicotine exceeds a certain threshold, the HPA axis may not be activated at the hypothalamic level, which may lead to a hyper-suppression of the HPA axis in habitual smokers in line with our observation [46]. Additionally, it was shown that habitual smokers have an attenuated response to the stress, which might be due to the stronger suppression of the HPA axis [46, 47]. As mentioned previously, body weight may affect the pharmacodynamics of dexamethasone. Smokers are generally leaner than non-smokers and difference in weight might affect the association between smoking status and cortisol suppression. However, our results remained essentially unchanged when the analysis was adjusted for weight.

None of the characteristics associated with the HPA axis suppression showed any association with morning cortisol levels alone, except instrumental disability. This suggests that the degree of cortisol suppression after the pharmacological stimulation cannot be inferred from basal cortisol levels.

Cognitive functions were related to the negative feedback of the HPA axis. Poor global cognitive functioning as assessed with the g-factor was related to low cortisol levels after dexamethasone intake at the age- and gender-adjusted model. However, this association was not significant in the multivariable model, in which analyses were adjusted for physical health and mental health items. Still, it is important to consider global cognitive functions as a possible determinant of the negative feedback control of the HPA axis in the general population. Also, instrumental disability, as an early indicator of cognitive problems [48], was associated with low cortisol levels after dexamethasone intake. Instrumental activities of daily living is a measure of the skills required to live independently and perform activities which are more complicated and higher-level tasks than in ADL such as shopping, managing finances, answering the telephone, preparing meals, or using transportation [49]. Disability in iADL often reflects severity of chronic, disabling diseases and is associated to decreased quality of life and disturbed social life [50, 51]. Moreover, iADL is disturbed earlier than ADL in people with cognitive problems [52] and it is used as a proxy of cognitive functioning. Further studies are needed to explore cognition in more detail in DST studies.

Symptoms of depression and major depressive disorders were not related to basal cortisol or suppression in linear models. Given the extensive evidence from clinical research on the relation between clinical depression and DST, there is little doubt that a certain percentage of depressed patients show non-suppression in the DST [53]. Results of the studies in outpatients or in the general population, however, vary largely [53–55]. In our study, we evaluated depressive symptoms in the general population, which may differ from the clinical populations in terms of severity of the disorder and socio-economical characteristics. Either, the depressive symptoms observed in this population have too little impact on HPA axis regulation, or the association is not uniform, i.e., depressive symptoms or disorders in some patients are related to a stronger and in others to less suppression of the HPA axis; this is possibly explained by subtypes of depression. Also, there is possibly a non-linear association between depression and the extremes of HPA axis functioning in the general population, which explains inconsistent results [56–58]. This is supported by an association of depressive symptoms with non-suppression of the HPA axis by very low doses of dexamethasone in the absence of an association along the continuum of suppression. Such a non-linear pattern was not observed for MDD most likely this was due to the small sample of persons with clinical depression.

Similarly, previous research on anxiety disorders and the negative feedback of the HPA axis provided conflicting findings, such as non-suppression [59, 60] and suppression [61, 62] in different anxiety disorders. These inconsistencies in the literature could stem from different physiological features of individual anxiety disorders, chronicity of the disorders and comorbid psychiatric diseases, mainly depressive disorders [62]. Similar to depression, there was a non-linear association between anxiety and the negative feedback control of the HPA axis. In our study, we found that having an anxiety disorder was related to the non-suppression of the HPA axis after dexamethasone intake. HPA axis abnormalities are also seen in psychotic patients. A stronger suppression of the HPA axis after dexamethasone intake has been reported in patients with psychosis [15]. In the current study, we assessed psychotic experiences indicating a very mild form of psychosis. Research exploring the association between the HPA axis functioning and psychotic experiences is very limited and is restricted to assessments of cortisol levels or stress reactivity rather than the negative feedback control of the HPA axis [63]. In the current study, we did not detect any associations between psychotic experiences and cortisol levels after dexamethasone intake.

In the main analyses, we did not find any associations between psychotropic medications and the cortisol levels after dexamethasone intake. However, psychotropic medication use was related to the enhanced suppression of cortisol after dexamethasone intake. Both antidepressants and antipsychotics have been related to increased cortisol suppression after dexamethasone intake previously [15, 64, 65] yet the underlying mechanisms remain unclear.

Our study had several strengths. First, this study had a large sample size, which is not common in studies using an interventional diagnostic test. Second, the population-based design of the current study increases the external validity of the results. Third, the very low-dose DST in this population-based study allowed us to detect subtle changes, which cannot be easily detected with a high dose DST in healthy people [7]. Fourth, the large sample size and extensive data collection of the study allowed us to evaluate several variables and determine which are the strongest predictors of the cortisol response after dexamethasone intake. Some limitations, however, should be considered. First, when used in outpatients or in the general population, the DST can suffer from the noncompliance to the dexamethasone protocol. Yet, noncompliance to dexamethasone intake was not very frequent in studies monitoring compliance, not even if participants with psychiatric disorders were included [5, 66]. Also, compliance in reporting the exact time of sampling could be a problem in the current study, as we did not use a saliva collection device recording time of sampling automatically. However, we instructed our participants only to return the saliva samples if they had taken the dexamethasone. We also emphasized the importance of recording the exact time of sampling. Participants were encouraged to report the true time of sampling to allow us to calculate the deviations from the planned times. As an indicator of compliance, the time interval between the first and second cortisol sampling was used in the sensitivity analyses. We detected no difference between compliers and non-compliers (according to an arbitrary definition of compliance) and therefore presented the results of the whole group to minimize selection bias. However, our results might suffer from residual information bias if noncompliance was not measured well. Second, our results cannot be generalized to the clinical psychiatric populations as coping styles, perceived stress and SES determinants may differ largely between clinical cohorts and general population. Third, we did not evaluate trauma history or PTSD, which might be an important determinant to evaluate [16–19]. However, the prevalence of PTSD is low. Moreover, obtaining a valid trauma history in older persons is challenging in the general population. Finally, testing non-linear associations with centiles increases the risk of type I error due to multiple testing. At the same time, it may lead to small subgroups, which reduce the statistical power Indeed, the lack of an association between MDD and non-suppression can most likely be attributed to the small sample size of persons with MDD in the current study.

## Conclusions

In this large population-based study, we found a consistent pattern of increased negative feedback control of cortisol using the very low-dose DST in females, smokers, less active persons, and those with low income and instrumental disability. In contrast, clinical and subclinical psychiatric and cognitive conditions were less important determinants than gender, low socioeconomic status and poor health. Prospective studies are needed to test to what extent increased suppression of cortisol is related to disease.

## Supporting Information

S1 File Supporting information. (DOCX)
