# Supplementary material for: The Very Low-Dose Dexamethasone Suppression Test in the General Population: A Cross-Sectional Study
Source: PLoS One. 2016 Oct 13;11(10):e0164348. doi: 10.1371/journal.pone.0164348 (PMC5063373; doi:10.1371/journal.pone.0164348)
Supplement: S1 File — (DOCX) [file pone.0164348.s001.docx]

Figure A. Distribution of cortisol levels before dexamethasone intake (*x*-axis represents categories of cortisol values and *y*-axis represents percentage of people within the cortisol range)

Figure B. Distribution of cortisol levels after dexamethasone intake (*x*-axis represents categories of cortisol values and *y*-axis represents percentage of people within the cortisol range)


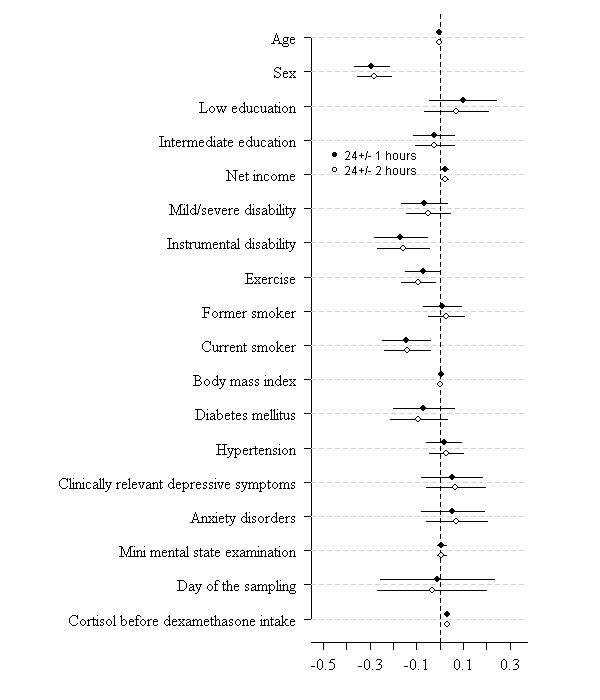


Figure C. Effect of compliance to saliva cortisol sampling on the association of different determinants with cortisol suppression after dexamethasone intake.

Figure C Legend: Regression coefficients and the confidence intervals of the associations between determinants and cortisol concentrations after dexamethasone intake in people with a time interval of 24 ± 1 hours (n=1632) between cortisol samplings and in people with a time interval of 24 ± 2 hours (n=1766). These estimates derived from the mutually adjusted model.

Table A. Relations of determinants with cortisol concentrations before dexamethasone intake

| **`** | **Cortisol concentration before dexamethasone intake** | | | |
| --- | --- | --- | --- | --- |
|  | **Mutually adjusted model** | | | |
| **Sociodemographic indicators** | *β* | B | 95% CI | *p* |
| Age (years) | 0.04 | 0.004 | -0.001; 0.01 | .14 |
| Sex (0=male, 1=female) | -0.03 | -0.04 | -0.10; 0.03 | .25 |
| Education |  |  |  |  |
| Low | -0.02 | -0.04 | -0.16; 0.08 | .54 |
| Intermediate | -0.04 | -0.05 | -0.13; 0.02 | .17 |
| High |  | (reference) | |  |
| Net income (ranked 1 to 13) | -0.002 | -0.01 | -0.02: 0.01 | .66 |
| **Health & lifestyle variables** |  |  |  |  |
| Mild/severe disability (0=no, 1=yes) | -0.04 | -0.07 | -0.16; 0.01 | .09 |
| Instrumental disability (0=no, 1=yes) | -0.06 | -0.12 | -0.22; -0.02 | .02 |
| Regular exercise (0=yes, 1=no) | -0.04 | -0.07 | -0.12; 0.004 | .07 |
| Smoking |  |  |  |  |
| Never smoker |  | (reference) | |  |
| Former smoker | 0.02 | 0.03 | -0.04; 0.09 | .46 |
| Current smoker | -0.02 | -0.04 | -0.12; 0.05 | .42 |
| Body mass index (kg /m^2^) | -0.09 | -0.01 | -0.02; -0.01 | <.001 |
| Diabetes mellitus (0=no, 1=yes) | 0.01 | 0.03 | -0.08; 0.14 | .54 |
| Hypertension (0=no, 1=yes) | -0.02 | -0.02 | -0.09; 0.04 | .51 |
| **Psychiatric problems & cognitive functions** |  |  |  |  |
| Depressive symptom score | -0.02 | -0.002 | -0.01; 0.003 | .50 |
| Clinically relevant depressive symptoms  (0=no, 1=yes) | -0.01 | -0.03 | -0.14; 0.08 | .62 |
| Major depressive disorder (0=no, 1=yes) | 0.04 | 0.18 | -0.07; 0.42 | .16 |
| Anxiety disorders (0=no, 1=yes) | -0.01 | -0.02 | -0.14; 0.10 | .75 |
| Mini Mental State Examination score | 0.07 | 0.03 | 0.01; 0.04 | .002 |
| Cognitive impairment (0=no, 1=yes) | -0.06 | -0.26 | 0.47; -0.04 | .02 |
| The *g*-factor of the cognitive tests | 0.06 | 0.04 | 0.003; 0.08 | .04 |
| Psychotic experiences (CAPE-positive items) frequency score | -0.06 | -0.30 | -0.65; 0.05 | .09 |
| Psychotropic medications (0=no, 1=yes) | -0.04 | -0.08 | -0.17; 0.01 | .08 |
| **Sampling variables** |  |  |  |  |
| Day of the sampling  (0=weekend, 1=weekday) | 0.00 | 0.00 | -0.21; 0.21 | .99 |
| Time of the sampling | -0.00 | -0.11 | -0.00; -0.00 | <0.001 |

*Abbreviations:* *β*, standardized beta; B, unstandardized beta; CI, Confidence Interval of the unstandardized beta; MMSE, Mini-Mental State Examination; CAPE, The Community Assessment of Psychic Experience

Table B. Relations of determinants with cortisol suppression categories

|  | Non-suppression  n=182 | | | Enhanced suppression  n=183 | | |
| --- | --- | --- | --- | --- | --- | --- |
|  | OR | 95 % CI | P | OR | 95 % CI | P |
| Depressive symptom score | 1.02 | 1.002; 1.05 | .03 | 1.002 | 0.98;1.02 | .85 |
| Clinically relevant depressive symptoms^*^ | 1.93 | 1.18; 3.17 | .01 | 1.05 | 0.63; 1.74 | .86 |
| Major depressive disorder^*^ | 1.51 | 0.50; 4.52 | .46 | 0.61 | 0.14; 2.68 | .51 |
| Anxiety disorders^*^ | 2.42 | 1.46; 4.00 | .001 | 0.72 | 0.39; 1.34 | .30 |
| Mini Mental State Examination score | 1.05 | 0.95; 1.17 | .35 | 0.98 | 0.91; 1.05 | .52 |
| Cognitive impairment^*^ | 1.51 | 0.44; 5.16 | .51 | 1.45 | 0.57; 3.67 | .43 |
| The *g*-factor of the cognitive tests | 0.97 | 0.79; 1.19 | .76 | 0.87 | 0.72; 1.04 | .13 |
| Psychotic experiences (CAPE-positive items) frequency score | 1.16 | 0.19; 7.13 | .87 | 1.59 | 0.28; 9.01 | .60 |
| Psychotropic medications^*^ | 1.55 | 0.99; 2.45 | .06 | 2.08 | 1.43; 3.02 | <.001 |

^*^0=no, 1=yes

**Instructions of Saliva Sampling**

Participants were instructed about the procedure. A detailed explanation of the reasoning of the test was given. Also, sampling times and time of dexamethasone intake were detailed and the importance of recording the exact time was emphasized. We informed participants about fasting and not brushing their teeth for 15 minutes before saliva sampling.

Participants were trained about using saliva-sampling devices, collecting saliva, storing them until the samples were collected. A dexamethasone pill was shown to the participants at the study center.

They were instructed to make an appointment as soon as they finish the procedure. Samples were either brought by participants or were collected by the study centre staff.
